# Fatal Hemorrhage from the Umbilical Cord Three Days after Birth

**Published:** 1848-01

**Authors:** 


*Fatal Hemorrhage from the Umbilical Cord three days after Birth.*—Dr. Keller stated on a recent occasion that he had been called upon to examine the subject of the following case of *fatal umbilical hemorrhage*, which recently occurred in the practice of a gentleman in town.

About half past one, P. M., on Tuesday, the 3d instant, Mrs. ——, aged 29, was delivered of her third, a fine plump male child. The infant continued quite well until five o'clock, when it began to vomit a quantity of green bilious matter. On Thursday morning, however, the child was apparently well, being able to suck greedily, but again vomited towards the afternoon. About two, A. M., on Friday morning, the mother first discovered that the binder, &c., of the child were "soaked with blood from the navel." (She stated that she had changed the linens of the infant about twelve P. M., and did not then notice the existence of any bleeding.) The practitioner who delivered her was immediately sent for, but could not visit at the time; he, however, told the parties to apply a ligature below the one that was already around the cord, but they did not *deem it their duty to interfere*, "but preferred waiting until the gentleman could find it convenient to come and tie the cord himself," which was not until between four and five o'clock, when he visited and examined the bleeding point which was at the root of the cord, and applied to it the nitrate of silver, which seemed at the time to be sufficient to prevent the farther continuance of the hemorrhage. Before half an hour had elapsed, however, he was again summoned, when he tied a ligature around the umbilicus, embracing a portion of the skin which formed its circumference. This treatment, however proved too late; the child died in a very few minutes after the application of the ligature.—*Monthly Jour. of Medical Science.*

